# Fascin1 expression in high-grade serous ovarian carcinoma is a prognostic marker and knockdown of fascin1 suppresses the proliferation of ovarian cancer cells

**DOI:** 10.3892/ijo.2013.2232

**Published:** 2013-12-30

**Authors:** SAE HYUN PARK, JI-YE SONG, YU-KYUNG KIM, JIN HYUNG HEO, HAEYOUN KANG, GWANGIL KIM, HEE JUNG AN, TAE HOEN KIM

**Affiliations:** 1 Department of Gynecologic Oncology, CHA Gangnam Medical Center, Gangnam-Gu, Seoul 135-907;; 2 Clinical Research Institute, CHA University, Bundang-Gu, Seongnam-Si, Gyeonggi-Do 463-712, Republic of Korea; 3 Department of Pathology, CHA Bundang Medical Center, CHA University, Bundang-Gu, Seongnam-Si, Gyeonggi-Do 463-712, Republic of Korea

**Keywords:** high-grade serous ovarian carcinoma, fascin1, prognosis

## Abstract

Fascin1 (*FSCN1*) involved in cell motility and filopodia assembly plays important roles in biological processes such as cancer invasion and metastasis of multiple epithelial tumors. High-grade serous ovarian carcinoma (HGSOC) is aggressive and metastatic by acquiring an invasive phenotype and this step requires remodeling of the actin cytoskeleton. Thus, the present study aimed to investigate the expression of fascin1 in HGSOC tissues as well as its clinical significance such as prognostic predictors and its utility of therapeutic target. Fascin1 and β-catenin were evaluated using immunohistochemistry on a tissue microarray of 79 HGSOC. Small interfering RNA (siRNA) approach was used to knock down fascin1 expression in ovarian cancer cell lines to determine whether fascin1 contributes to tumor cell proliferation, migration and invasion. Fascin1 expression levels were determined by western blot analysis after siRNA transfection using two human ovarian cancer cell lines (SKOV3 and OVCAR3). Fascin1 overexpression was significantly correlated with lymph node involvement, distance metastasis and high International Federation of Gynecology and Obstetrics (FIGO) stage (III/IV) (P<0.05). A Kaplan-Meier analysis showed that the fascin1 expression group was significantly associated with poor overall survival (P=0.010). We showed that inactivation of fascin1 by siRNA transfection led to a drop in cell viability, and significantly decreased tumor cell proliferation, migration and invasiveness compared to untransfected cells. We found that fascin1 expression is a potential poor marker of prognosis for patients with HGSOC and knockdown of fascin1 suppresses ovarian cancer cell proliferation and migration, this could be applied for therapeutic targets in ovarian cancer treatment.

## Introduction

It has been estimated that nearly 22,280 new cases of ovarian cancer were diagnosed in the US in 2012, with approximately 15,500 people dying of the disease (1). Ovary cancer incidence in Korea was similar to that in women worldwide and ovarian cancer-related mortality rates has been increasing (2). High-grade serous ovarian carcinoma (HGSOC) is the most lethal form of gynecologic cancer and mostly advanced clinical stages (FIGO stages III and IV) of the disease at the time of diagnosis, and they exhibit more metastatic lesions. HGSOCs are heterogenous group of diseases involving many different tumorigenic pathways and harboring a variety of genetic mutations. It seems that the understanding of the possible mechanisms underlying the aggressive progression of HGSOC is required to elaborate their fatal clinical outcome. It has been suggested that cancer cells become aggressive and metastatic by acquiring an invasive or meta-static phenotype. We suggests this step requires remodeling of actin cytoskeleton and fascin1 fundamentally contributes to disease progression and patients prognosis.

Fascin1 (gene name *FSCN1* in human) is a 55 kDa actin-bundling protein and is an important regulatory element in the maintenance and stability of parallel bundles of filamentous actin in a variety of cellular contexts (3). This can help form the cellular structures critical to cell movement, such as filopodia and spikes, and its depletion by small interfering RNA (siRNA) leads to a substantially reduced number of filopodia (4). It is not clear how fascin1 promotes invasive motility in cancer cells, but Li *et al* recently provided new evidence that fascin1 stabilizes actin bundles in invasive foot structures termed invadopodia, and they suggested that fascin1 is an integral component of invado-podia and it is important for the stability of actin (5). Thus, fascin1 provides cells with powerful invasive properties that may confer increased metastatic potential. Fascin1 has recently received considerable attention as a new biomarker or potential therapeutic target because it is absent or at very low levels in epithelial cells (6), whereas the overexpression of fascin1 has been reported in tumors of the lung (7), esophagus (8), breast (9), colon (10), urinary bladder (11) and ovary (12) usually correlating with high grade, extensive invasion, distance metastasis and poor prognosis. The aberrant expression of fascin1 in these cancers has been indicated to be linked to increased cell motility and tumor invasiveness. Hashimoto *et al* hypothesized that fascin1 upregulation is generally correlated with the aggressive behavior of cancer cells, independent of the tissue origin (3). Depletion of fascin1 reduced penetration into reconstituted matrix and greatly reduced the spikeness of invading cells. Thus, fascin1 appears to provide cancer cells with stable long lived invasive protrusion that allow them to invade into the surrounding matrix. Previous studies of fascin1 expression in ovarian cancer suggested that upregulation of fascin1 in tumor tissue may promote invasion of ovarian carcinoma (12). Similarly, fascin1 expression of advanced colorectal adenocarcinomas correlated with shorter survival in stage III/IV patients (10). Although the hypothesis of regulation by β-catenin signaling has received attention, how fascin1 transcription is activated in carcinoma cells is largely unknown. Dysregulation of the WNT/β-catenin signaling pathway has been implicated in tumorigenesis at several sites including the colon, rectum, breast, liver and ovary. Fascin1 has been shown to bind with β-catenin at leading cell edges and cell-cell borders supporting its role in modulating the functions of cell motility and adhesion (13). Because β-catenin has been reported as a second binding partner for fascin1, we examined the expression of β-catenin in HGSOC and on the possible association with fascin1. It was reported that in ovarian cancer, nuclear localization of β-catenin was observed in 23% of serous tumors (14). Knockdown of fascin1 in human colon carcinoma cells results in decreased adhesion dynamics and subsequent reduction in cell migration (15). In support of this, a number of recent studies have shown clear roles for fascin1 in mouse models of tumor formation. Two reports demonstrated that colon carcinoma cells stably expressing shRNA to knockdown fascin1 showed significantly decreased tumor growth and development in xenograft mouse models (4,15). Animal studies have reported a positive correlation between fascin1 expression and tumor invasiveness (16). Expression of fascin1 positively correlates with clinically aggressive tumors and as such has recently received considerable attention as both a potential prognostic marker and therapeutic target for treatment of metastatic disease (17).

The aims of this study were to determine the expression of fascin1 and β-catenin in HGSOC and to correlate its expression with clinicopathologic parameters and demonstrate the possibility of prognostic predictors. In addition, we investigated the effects of fascin1 on cancer cell proliferation, migration and invasion of ovarian cancer cells to determine the potential role of fascin1 in ovarian cancer progression and utility of therapeutic target. We propose that fascin1 promotes invasion and metastasis of HGSOC cells and is associated with a more aggressive phenotype and poor clinical outcome.

## Materials and methods

### Case selection and tissue preparation for fascin1 and β-catenin immunohistochemistry

The cases were collected at the CHA Bundang Medical Center, School of Medicine, CHA University from 1998 to 2011. A total of 79 patients with HGSOC were enrolled in this study, and their clinicopathological characteristics are summarized in Table I. The ages ranged from 24 to 83 years (median age, 54 years); 37 patients (46.8%) were ≥55 years and 42 patients (53.2%) were <55 years old. The FIGO stages at initial diagnosis were as follows: low stage (I/II) in 18 cases (22.8%) and high stage (III/IV) in 61 cases (77.2%). Lymph node involvement and distant metastasis were detected in 45 (56.9%) and 24 cases (30.4%), respectively. The mean follow-up interval was 75.9 months (range, 2–139 months). The patients were treated with a first-line chemotherapeutic regimen consisting of paclitaxel and cisplatin or carboplatin after radical surgery.

All cases were reviewed by two pathologists. Tissue cores for tissue microarray were collected from tumor (primary site) and control sections (normal fallopian tube and benign serous tumor). Tissue microarrays were constructed from archival formalin-fixed, paraffin-embedded tissue blocks using a manual tissue arrayer (Quick-Ray Manual Tissue Microarrayer; Unitma Co. Ltd., Seoul, Korea). Tissue cylinders with a diameter of 3 mm were punched from the tumor area of the donor block and were transferred to a recipient paraffin block. Tissue microarrays were sectioned to a 4-*μ*m thickness.

### Immunohistochemistry

Tissue microarray sections were dewaxed in xylene, rehydrated in alcohol and immersed in 3% hydrogen peroxide for 10 min to suppress endogenous peroxidase activity. Antigen retrieval was performed by heating (100°C) each section for 30 min in 0.01 mol/l sodium citrate buffer (pH 6.0). After three rinses in phosphate-buffered saline (PBS) for 5 min, each section was incubated for 1 h at room temperature with mouse monoclonal antibodies to human fascin1 (Epitomics, Burlingame, CA, USA; 1:100), and human β-catenin (Novus Biologicals, Littleton, CO, USA) all diluted in PBS; washed three times in PBS for 5 min; incubated with horseradish peroxidase-labeled rabbit anti-mouse immunoglobulin (DAKO; 1 h at room temperature); washed three times; and incubated with a solution of diaminobenzidine (DAB) at room temperature to visualize peroxidase activity.

### Evaluation of immunostaining

Two experienced pathologists evaluated the immunoreactivity and histological appearance of all tissue samples in the microarray. The intensity of cytoplasmic tumor cell staining was scored on a scale of 0 to 3: with 0 (no staining), 1 (weak intensity), 2 (moderate intensity) and 3 (the strongest intensity), and extent of tumor cells with cytoplasmic staining at each intensity was estimated. The extent of staining was scored as 0 (0%), 1 (1–25%), 2 (26–50%), 3 (51–75%) or 4 (76–100%), according to the percentages of positively stained areas in relation to the whole carcinoma area. The final scores for fascin1 immunostaining was obtained by mutiplying the staining intensity and the extent scores, score ranging from 0 to 12. We designated as positive in the cases with final staining score of ≥3.

### Cell lines, media and culture conditions

The human ovarian cancer cell lines (SKOV3, OVCAR3) were purchased from the American Type Culture Collection (Rockville, MD, USA). OVCAR3 cells were cultured in RPMI-1640 medium containing 10% fetal bovine serum (FBS), 100 U/ml penicillin and 100 mg/ml streptomycin. SKOV3 cells were cultured in McCoy’s 5A medium supplemented with 10% FBS, 100 U/ml penicillin and 100 mg/ml streptomycin. Cell lines were incubated at 37°C in a humidified atmosphere consisting of 5% CO_2_ and 90% humidity. These cell lines grew in a monolayer and were passaged when cultures were 70–80% confluent.

### Small interfering RNA preparation and transfections

Cells were plated at 70% confluency in McCoy’s 5A, RPMI-1640 containing 10% serum without antibiotics. We diluted 200 pmol/ml siRNA into 500 *μ*l serum-starved media (Gibco, Grand Island, NY, USA) without antibiotic-antimycotic (Invitrogen, Carlsbad, CA, USA) solution. We diluted 10 *μ*l Lipofectamine 2000 (Invitrogen) into 500 *μ*l of the above described media, incubated for 5 min at room temperature, and added 500 *μ*l of diluted transfection mixture containing the FSCN1 siRNA for another 20 min at room temperature. The transfection complex mixture was added to the cells. Scrambled siRNA with Lipofectamine 2000 alone was used as control. After 6 h, the medium was changed, and the samples were assayed after 24, 48 and 72 h until ready for further assay.

### RNA isolation and quantitative real-time PCR for FSCN1 expression

Total RNA was extracted from fresh tissues and the ovarian cancer cell lines SKOV3, and OVCAR3 were homogenized in TRIzol reagent (Invitrogen) in accordance with the manufacturer’s instructions. RNA purity and concentration were confirmed by spectrophotometry using a NanoDrop ND-1000 instrument (NanoDrop Technologies, Wilmington, DE, USA). First-strand cDNA synthesis was performed using a Superscript III kit (Invitrogen). cDNA samples were analyzed in triplicate using the Bio-Rad CFX96 Real-Time PCR Detection System. Briefly, 1 *μ*g of total RNA was amplified using the TaqMan Gene Expression Assay (Applied Biosystems, Paisley, UK) for the analyses of GAPDH (ABI code: Hs99999905_m1), and FSCN1 (ABI code: Hs00979631_g1), respectively. The PCR reaction mix had a volume of 20 *μ*l and contained 10 *μ*l 2X TaqMan master mix (Applied Biosystems), 1 *μ*l primer and probe kit (Applied Biosystems), 1 *μ*l cDNA and 8 *μ*l of diethylenepyrocarbonate (DEPC) water. The reverse transcription conditions used were as follows; 50°C for 2 min, 95°C for 10 min, followed by 40 cycles of 95°C for 15 sec and 60°C for 1 min. RNA levels were quantified at least three times. Transcript levels were normalized versus GAPDH expression, and gene expression was calculated using 2^−ΔΔCt^.

### Western blot analysis

Cells were homogenized and extracted with protease extraction buffer (Pro-Prep, iNtRON Biotechnology, Korea) and centrifuged at 4°C, 13,000 rpm, for 15 min. Protein concentration were determined by the Bradford assay. Total proteins were electrophoresed on 10% Sodium Dodecyl Sulfate-Polyacrylamide gel. Separated proteins were transferred onto nitrocellulose membrane at 100 V for 2 h, and the membranes were blocked in 5% milk for 1 h. The membrane was incubated with 1:1,000 dilution of rabbit anti-FSCN1 (Epitomics), β-actin (1:10,000, Santa Cruz Biotechnology, Santa Cruz, CA, USA) overnight at 4°C, washed with TBST and incubated with 1:5,000 goat anti-rabbit IgG, 1:5,000 rabbit anti-mouse IgG (Santa Cruz Biotechnology) secondary antibodies (1:5,000) for 1 h at room temperature. After the membrane had been washed with TBST, the protein bands were visualized using ECL reagent (iNtRON Biotechnology). The quantification of protein was done by densitometric digital analysis of protein bands using Quantity One^®^ 1-D Analysis Software version 4.6.7 (Bio-Rad Laboratories, Hercules, CA, USA). Each protein band was normalized to the corresponding β-actin band.

### Wound-healing assay

Cell migration was measured using an *in vitro* wound-healing assay. Cells were allowed to form a confluent monolayer in a 96-well tissue culture plates coated with gelatin before wounding. The wound was created by scraping monolayer cells with a sterile pipette tip across the monolayer. The wounded monolayers were washed twice with PBS to remove cell debris. Monolayers were incubated in cell culture medium and imaged through a microscope and photographed with a digital camera (CoolPix 950; Nikon) at 24 h.

### Colony-forming assay

Cells (SKOV3, OVCAR3) were seeded at 1×10^5^ cells per well in 6-well plates. The next day, cells were transfected with fascin1 siRNA and incubated for 48 h. Transfected cells were then replated at 300 cells per well in 6-well culture dishes. After 14 days, colonies were visualized using hematoxylin after fixation with 4% paraformaldehyde for 10 min and then counted. Groups of >50 cells were scored as colonies.

### Matrigel invasion assay

Cell invasion assay was carried out using Boyden chambers containing Transwell (Corning costar #3422) membrane filter inserts with pore size of 8 *μ*m. The Transwell membrane was coated with Matrigel at 3:7 dilution (BD Biocoat, Bedford, MA, USA) for invasion assay, cells (1×10^4^) in McCoy’s 5A medium containing 0.1% BSA were seeded on Boyden chambers (upper chamber). The lower chambers were filled with McCoy’s 5A medium containing 10% FBS. After 48 h of invasion at 37°C, cells passing through the filters into bottom wells were fixed in 100% ethanol and stained with hematoxylin and eosin (Sigma-Aldrich, St. Louis, MO, USA).

### Statistical analysis

SPSS 19.0 software was used for statistical analysis (Chicago, IL, USA). The χ^2^ test and Fisher’s exact test were used for comparison of the variables. Survival curves were estimated by the Kaplan-Meier method and compared by the log-rank test. Univariate and multivariate analyses were based on the Cox proportional hazards model. Wound healing, colony forming, and invasion assays were analyzed using a one-way analysis of variance. For all analyses, P-values <0.05 were considered statistically significant.

## Results

### The association between immunoreactivity of fascin1 expression and clinical parameters in patients with HGSOC and evaluation of β-catenin immunostaining

**Figure 1.**
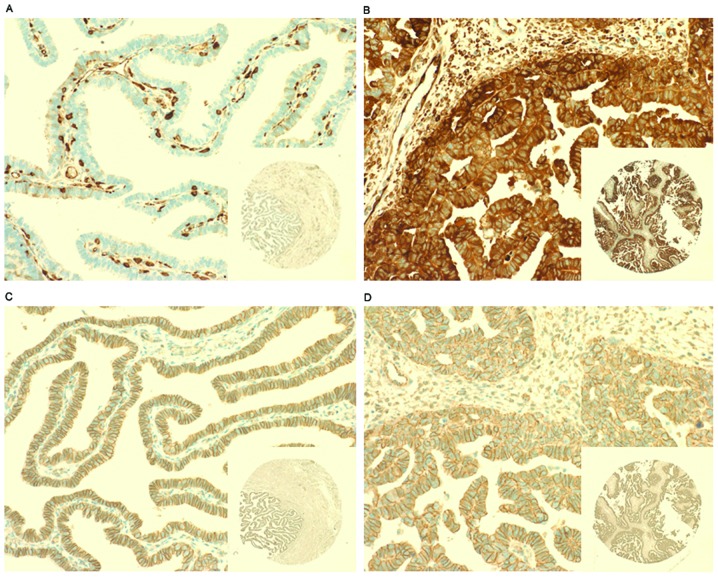
Representative immunohistochemical analysis of the fascin1 and β-catenin expression. (A) Fascin1 was negative in the epithelial cells of the fallopian tube (negative control) and immunopositive in the endothelial cells (positive control) and (B) positive staining in cytoplasm of tumor cells of the high-grade ovarian serous carcinoma. β-catenin was observed clear membrane staining in all the cases of (C) fallopian tube and of (D) high-grade ovarian serous carcinoma.

To investigate whether fascin1 expression is related to HGSOC, we analyzed fascin1 expression in 79 HGSOC tissues. Fascin1 was immunonegative in the epithelial cells of fallopian tube as a negative control and immunopositive in the endothelial cells as a positive control (Fig. 1A). Fascin1 positive immunostained cells were those containing dark brown granules mainly distributed in the cytoplasm of HGSOC cells (Fig. 1B). This finding demonstrates upregulation of fascin1 as a phenotypic alteration in HGSOC cells. Cells with positive β-catenin expression were defined as those cells containing dark brown nuclei or cytoplasmic staining, but there is no significant β-catenin positive staining cells, whereas all cancer cells and fallopian tube epithelial cells (normal control) in this study showed membrane staining (Fig. 1C and D). Table I shows the association between fascin1 expression and clinicopathological parameters. We found that fascin1 overexpression was significantly correlated with high FIGO stage (III/IV) (P=0.021), lymph node involvement (P=0.034) and distant metastasis (P=0.040). However, fascin1 expression was not associated with other parameters such as age, and tumor recurrence.

### Fascin1 expression is an independent prognostic marker in patients with high grade ovarian serous carcinomas

**Figure 2.**
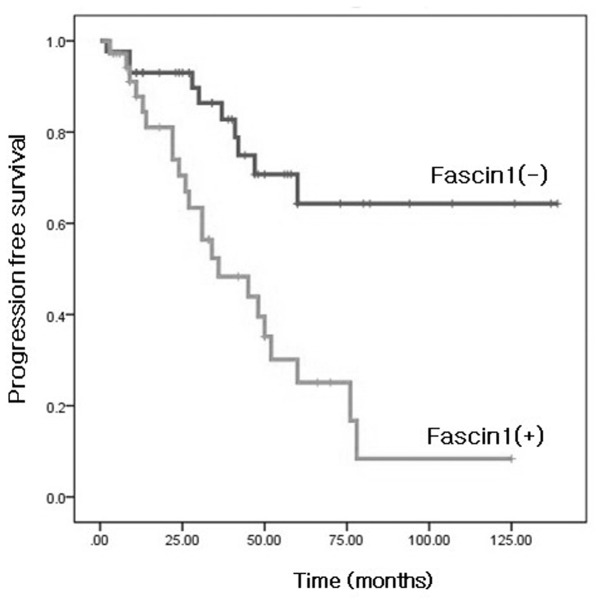
Kaplan-Meier survival analysis of progression-free survival in all patients according to fascin1 expression. Significant differences among the subgroups with positive (dimed line) and negative (bold line) fascin1 expression indicate poor outcomes in patients with fascin1 expression group. Fascin1 expression group was significantly correlated with shorter progression-free survival (P<0.001). The log-rank test yielded significant P-values.

We also evaluated whether fascin1 immunoreactivity predicted survival of patients with HGSOC. A Kaplan-Meier survival curve showed that the fascin1 expression group was significantly associated with poor survival of the patients (Fig. 2). The univariate and multivariate analyses results of progression-free survival for HGSOC are shown in Table II. The results of univariate analysis showed that age (P=0.012), FIGO stage (P=0.010), distant metastasis (P<0.001), and fascin1 expression (P<0.001) were correlated with progression-free survival. A multivariate Cox regression analysis revealed that age (P=0.030), and fascin1 expression (P=0.008) were independent prognostic factors of progression-free survival.

### Fascin1 siRNA inhibits the expression of fascin1 in ovarian cancer cell lines

**Figure 3.**
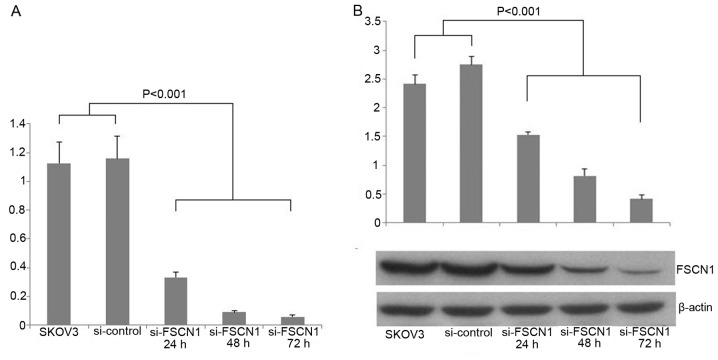
Fascin1 expression after siRNA transfection. (A) Real-time PCR analysis revealed the fascin1 expression was inhibited in the cancer cells transfected with fascin1 siRNA compared with control cancer cells (P<0.001). (B) Western blot analysis showed fascin1 siRNA inhibited fascin1 expression compared with control cancer cells. β-actin was detected as a loading control (P<0.001).

We used siRNA against fascin1 to transfect the SKOV3 ovarian cancer cells. We examined fascin1 mRNA expression compared with control cells after transfection. The result showed that fascin1 mRNA expression was reduced by 71.3, 92.3 and 95% of the transfected cells with 24, 48 and 72 h, respectively (P<0.001; Fig. 3A). To demonstrate the efficiency of fascin1 silencing at the protein expression level, western blot analysis was used to detect the fascin1 protein expression levels in 24, 48 and 72 h after transfection. We found that fascin1 expression reduced by 44.3, 70.2 and 85.1% of the transfected cells with 24, 48 and 72 h, respectively, compared with control cell line (P<0.001; Fig. 3B).

### Effects of fascin1 inactivation on cancer cell migration, proliferation and invasion activity in fascin1 siRNA transfected ovarian cancer cell lines

**Figure 4.**
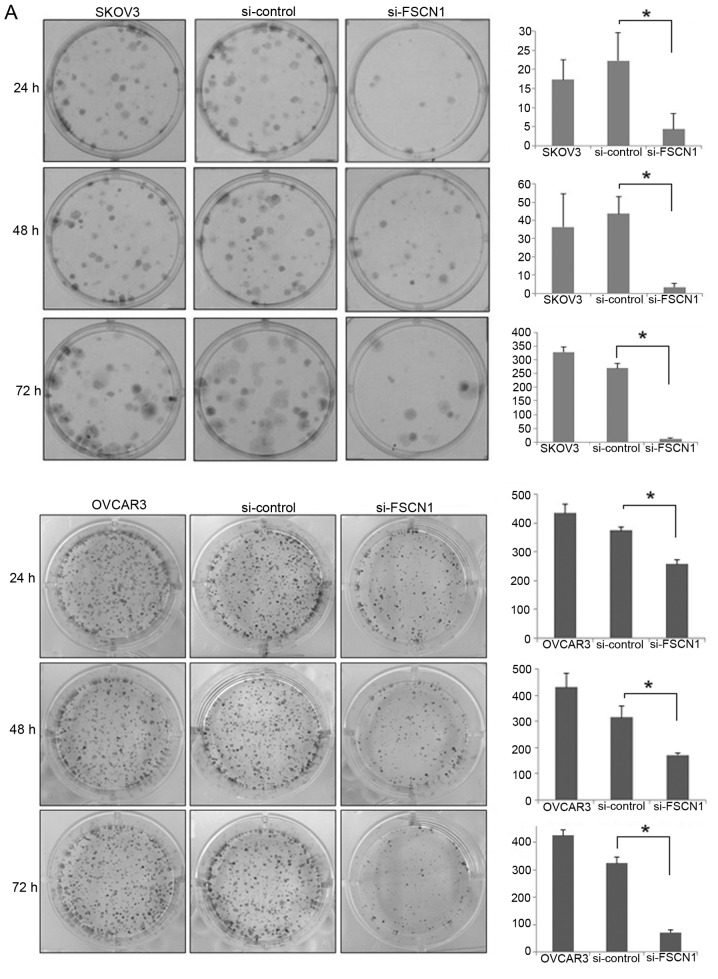

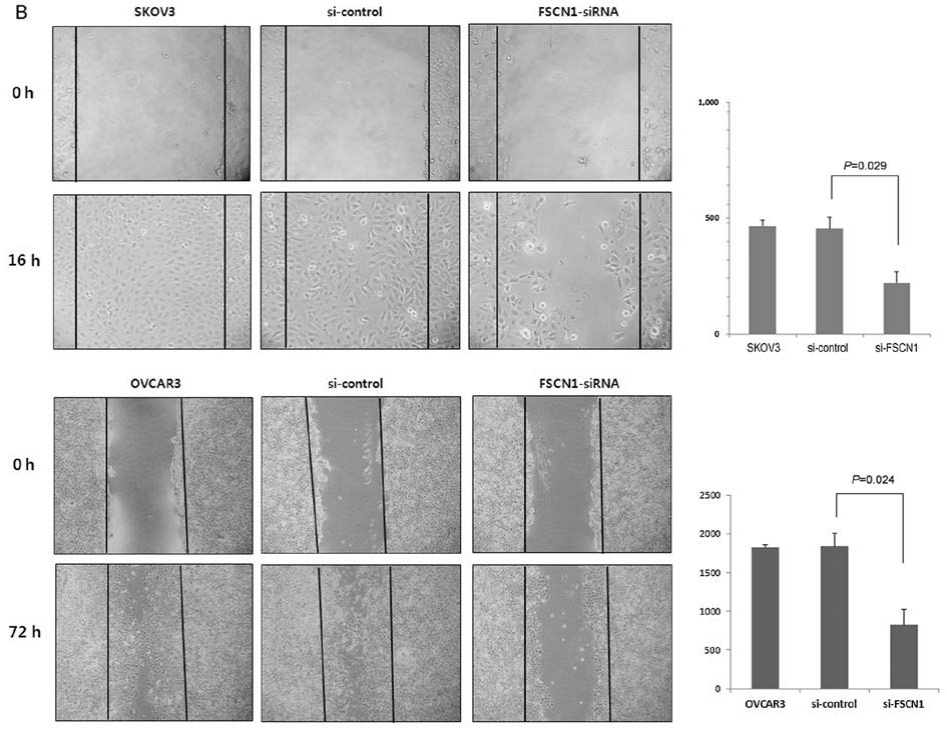

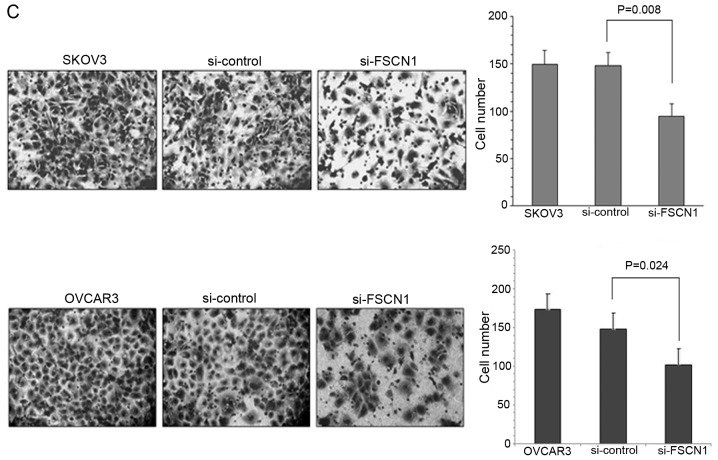
The inactivation of fascin1 inhibits proliferation and invasive ability of ovarian cancer cell lines SKOV3, and OVCAR3. (A) Cell proliferation was measured by colony forming assay after fascin1 siRNA transfection. The colony formation of the transfected cells grew significantly slower than control cells at 72 h. Colony numbers of transfected cells decreased significantly to 95.7% (SKOV3), 78.1% (OVCAR3) compared with that in control cells (72 h) (P<0.05). (B) Wound-healing assays were performed to examine the effect of fascin1 inactivation on cell migration (×100). The migration activities of the transfected cells were decreased 51.3% (SKOV3), and 55.3% (OVCAR3) compared to control cells at 16 h (P<0.05). (C) Matrigel invasion assays were performed to examine the effect of fascin1 on cell invasion (×100). Diagram of the cell count in the membrane. Fascin1 siRNA transfected cells led to 35.8% (SKOV3), and 31.1% (OVCAR3) decrease in the number of invasive cells (P<0.05). The above results are from 3 independent experiments.

We performed wound healing, colony forming and Matrigel invasion assays after fascin1 siRNA transfection. Colony numbers of transfected cancer cells decreased significantly to 95.7% (SKOV3), 78.1% (OVCAR3) compared with that of control cells at 72 h (P<0.05; Fig. 4A). Cell motility following wound generation showed less cell migration in transfected cells compared with that of control cells (P<0.05; Fig. 4B). After 16 h, we observed that transfected cells resulted in 51.3% (SKOV3), and 55.3% (OVCAR3) decreased migrating cell numbers in comparison with that of the control. The Matrigel invasion assay was used to assess the invasiveness of the cancer cells. The staining results are shown in Fig. 4C at 48 h. The control cells were more invasive and fascin1 siRNA transfected cells decreased significantly to 35.8% (SKOV3), 31.1% (OVCAR3) compared with that of control cells (P<0.05).

## Discussion

Fascin1 has received great attention as a potential therapeutic target among cytoskeletal proteins because multiple clinical studies have implicated its expression correlates with poor prognosis and metastasis in multiple carcinomas. This may be because fascin1 is not normally expressed in some epithelial tissues and when it is upregulated as a part of a mechanism of cancer cell epithelial to mesenchymal progression, it confers special motility and invasive properties on cancer cells (18). Given that fascin1 plays a key role in assembly and stability of actin-rich bundles within protrusive structures in cancer cells, it is possible that upregulation of fascin1 in metastatic disease *in vivo* can assist in promoting cell invasion through cytoskeletal assembly. A further study identified fascin1 as being highly upregulated in a subpopulation of circulating human breast tumor cells in a xenograft model that undergo re-colonization of their tumors of origin in a process termed ‘self-seeding’ (9). Upregulation of fascin1 in tumoral tissue may promote invasion of ovarian carcinoma by cell-matrix adhesion (19). It has been reported that fascin1 was not expressed in epithelial cells of normal fallopian tube and benign serous tumor but overexpressed in ovarian serous carcinoma (12,20,21). Therefore, the expression of fascin1 has been shown to be associated with invasive phenotype and poor prognosis in ovarian serous tumor. It was reported to be highly upregulated human cancers suggesting that fascin1 may fundamentally contribute towards disease progression (15). This is one of the reasons that fascin1 has received considerable attention recently as an emerging key prognostic marker of metastatic disease. We are currently expanding our study to evaluate the prognostic significance of fascin1 expression and its effect of invasiveness in patients with HGSOC. We found that with the exception of a few specimens, whereas fascin1 was not detected in the normal fallopian tube and benign serous tumor, the expression of fascin1 was significantly elevated in HGSOC tissue, and this increase was FIGO stage-dependent. We also demonstrated that fascin1 expression was higher in patients with lymph node involvement and distant metastasis, this results showed that fascin1 is a possible marker for predicting distant metastasis of HGSOC. We conclude that fascin1 expression correlates with invasiveness of HGSOC and the presence of fascin1 in primary tumors has predictive value in determining the advanced clinical stage. Consistent with our findings, Kabukcuoglu *et al* demonstrated that fascin1 expression was correlated with clinical stage, especially higher in tumors than normal samples (19). Our results also demonstrated fascin1 expression have a strong influence on patients survival outcome. Daponte *et al* reported that strong fascin1 expression is an independent prognostic factor for survival of advanced ovarian serous carcinoma (22). Compatible with this report, our study also demonstrated that fascin1 expressing group was significantly associated with shorter progression-free survival. A multivariate analysis showed that fascin1 expression was an independent negative prognostic factor for progression-free survival. Taken together, we suggest that fascin1 overexpression is an indicator of poor prognosis in patients with HGSOC.

The precise mechanism of fascin1 has not been clearly elucidated. In some cases, high fascin1 expression has been correlated with low E-cadherin expression, indicating that as cells progress through the epithelial to mesenchymal transition (EMT), they gain fascin1 whilst losing E-cadherin. There is evidence that fascin1 expression is regulated by two pathways, the WNT activated TCF/LEF (T cell factor/lymphocyte enhancer-binding factor) transcriptional signaling pathway that promotes EMT and cyclic-AMP response element binding protein (CREB) and the arylhydrocarbon receptor (AhR) (16,23). Hashitomoto *et al* suggest that upregulation of fascin1 in aggressive human carcinomas appears to have a multi-factorial basis but CREB and AhR as specific regulators of fascin1 transcription do not support the hypothesis that β-catenin signaling has a central role (23). The critical component of WNT signaling pathway, β-catenin, plays an important role in this process. The aberrant activation of β-catenin-TCF signaling eventually leads to the accumulation of β-catenin in the nucleus, where it forms transcriptionally active complexes with TCF/LEF (24). It has been recognized that fascin1 binds to β-catenin at leading cell edges and cell-cell border as a novel target of β-catenin-TCF signaling, supporting its role in modulating the functions of cell motility and adhesion and fascin1 expression is tightly regulated during development of colon cancer metastasis (16). However, Jawhari *et al* demonstrated that fascin1 overexpressed cells were not affected in their ability to localize E-cadherin and β-catenin to cell-cell margin (25). It has also been reported that fascin1 was a target of the β-catenin pathway in the invasive progress of ovarian cancer. Gamallo *et al* reported that β-catenin nuclear localization was correlated with improved survival in early stage ovarian carcinomas, while Lee *et al* revealed that 23% positivity of β-catenin nuclear localization in HGSOC and they suggested that it was one of the mechanisms for tumorigenicity in HGSOC possibly through activation of the TCF/β-catenin pathway (14,26). These results were not compatible with our findings. Otherwise, Cho *et al* demonstrated that nuclear expression of β-catenin was detected in only one case (0.6%) of serous ovarian tumor, membrane staining was not different among benign, borderline, and malignant tumors, and Kildal *et al* also reported that they observed β-catenin nuclear localization in only one of 59 (1.7%) serous adenocarcinoma cases (20,27). If ovarian serous tumor follows this pathway, nuclear localization of β-catenin is increased due to the decrease of contact inhibition of E-cadherin/β-catenin, but in this study, nuclear localization of β-catenin was not observed. Whereas all cases of HGSOC in our study demonstrated β-catenin membrane staining, no detectable β-catenin nuclear or cytoplasmic staining was found. Therefore, we suggest that β-catenin nuclear expression may be an uncommon finding in HGSOC. Thus, due to a limited number of studies and different results, further investigation including evaluation of TCF activity of WNT signaling pathway in HGSOC is required.

Fascin1 is currently the only actin bundling protein localized along the entire length of filopodia and its depletion by small interfering RNA (siRNA) leads to a substantially reduced number of filopodia (4). The fascin1 in invadopodia appears to stabilize the actin, as knockdown of fascin1 increases the mobile fraction in invadopodia and decreases the lifetime and size of invadopodia (5). Depletion of fascin1 reduced penetration into reconstituted matrix and greatly reduced the spikeness of invading cells. Thus, fascin1 appears to provide efficient stable invasive protrusions that allow them to invade into the matrix. Given the fact that there is a role for fascin1 in cancer cell migration and proliferation, we examined the effects of fascin1 on cell proliferation and invasion measured by colony forming, wound healing and Matrigel-coated Transwell invasion assays. To demonstrate the functional effect of fascin1 on invasiveness of ovarian serous carcinoma, we performed fascin1 siRNA study, which provided us the functional consequences of fascin1 inactivation. It has been shown that fascin1 downregulation had inhibitory effects on tumor cell migration, proliferation, and invasiveness of esophageal squamous cell carcinoma cell lines, suggesting that fascin1 contributes to tumor progression and could be a therapeutic molecular target (28). Hu *et al* have reported that the expression of fascin1 in ovary tumor cell cultures is significantly associated with the ability to grow and spread intraperitoneally after intraperitoneal inoculation supporting the role of fascin1 in ovarian metastasis (29). Consistent with this report, our study further demonstrated that inactivation of fascin1 by siRNA technique resulted in a decreased proliferation, motility and invasiveness of ovarian cancer cells *in vitro*, suggesting that fascin1 contributed to the invasion and metastasis of HGSOC. Thus, these results imply that fascin1 expression in human ovarian cancer cells plays an important role in their motile, invasive, and metastatic capacities. Hashimoto *et al* suggested that suppression of fascin1 expression using siRNA resulted in fewer filopodia, altered cell protrusions, decreased Rac-dependent migration on laminin, and decreased turnover of focal adhesions (15). Chen *et al* has reported that fascin1 as the primary target for the antitumor agent migrastatin, macroketone in its inhibition of tumor cell migration, invasion and metastasis (30). The data demonstrated that migrastatin binds to the actin-binding site in fascin1 and thus inhibits fascin1-dependent invasion *in vivo*. The identification of such a specific inhibitor of fascin1-actin binding will additionally provide new molecular targets for cancer treatment.

In conclusion, we have demonstrated that fascin1 expression was significantly correlated with advanced clinical stage and related to survival of the patients. Our results support the concept that the blockage of fascin1 influences ovarian cancer cell proliferation and invasive potential. Our findings highlight the important role of fascin1 in aggressive progression of HGSOC and imply that fascin1 is a potential prognostic marker for patients with HGSOC and suggesting its use as a potential therapeutic target for ovarian cancer treatment.

## Figures and Tables

**Table I.** The association between fascin1 expression and clinicopathologic parameters of patients with high-grade ovarian serous carcinoma.

Parameter	Case n=79	FSCN1 expression		P-value
		Negative (%)	Positive (%)	
Age, years				
<55	43	26 (60.5)	17 (20.1)	0.116
≥55	36	16 (44.4)	20 (55.6)	
FIGO stage				
Low (I/II)	18	14 (77.8)	4 (22.2)	0.021^a^
High (III/IV)	61	29 (47.5)	32 (52.5)	
LN involvement				
No	34	23 (67.6)	11 (32.4)	0.034^a^
Yes	45	20 (44.4)	25 (55.6)	
Distant metastasis				
No	55	34 (61.8)	21 (38.2)	0.040^a^
Yes	24	9 (37.5)	15 (62.5)	
Recurrence				
No	52	29 (55.8)	23 (44.2)	0.462
Yes	27	14 (51.9)	13 (48.1)	

LN, lymph node; SD, standard deviation;

a statistical significance.

**Table II.** Univariate log-rank analysis and multivariate Cox regression analyses of progression-free survival (months, mean ± standard deviation) in patients with high-grade ovarian serous carcinoma.

Variable	Case	No. of deaths	Progression-free survival	P-value	Progression-free survival hazard ratio (95%CI)	P-value
Age, years						
<55	42	12	93.2±10.3	0.012^a^	2.412 (1.086–5.354)	0.030^a^
≥55	37	20	49.7±6.7			
LN involvement						
Absent	37	11	78.1±10.1	0.148	1.749 (0.731–4.185)	0.209
Present	42	21	69.2±9.6			
FIGO stage						
I/II	18	1	97.5±8.6	0.010^a^	3.945 (0.475–32.792)	0.204
III/IV	61	31	67.6±7.7			
Distant metastasis						
Absent	58	15	94.2±9.1	<0.001^a^	1.803 (0.879–3.737)	0.113
Present	21	17	43.9±8.6			
FSCN1 expression						
Negative	43	10	102.1±9.7	<0.001^a^	2.955 (1.32–6.60)	0.008^a^
Positive	36	22	46.3±6.7			

LN, lymph node; CI, confidence interval;

a statistical significance.
